# Society Meetings

**Published:** 1902-07

**Authors:** 


### SOCIETY MEETINGS.

THE American Association of Obstetricians and Gynecologists will hold its fifteenth annual meeting at The Raleigh, Washington, D.C., Tuesday, Wednesday and Thursday, September 16, 17 and 18, 1902, under the presidency of Dr. Edwin Ricketts, of Cincinnati, O. A list of forty papers has been offered, and a meeting of great interest is in prospect. Members desirous of securing rooms should address Mr. T. J. Talty, manager, The Raleigh, Washington, D.C.

THE Association of Military Surgeons of the United States held one of the most successful meetings—its eleventh annual—at Washington, D. C., June 5-7, 1902, under the presidency of Lt.-Col. John Van Rensselaer Hoff, Deputy Surgeon-General U. S. Army. The program was filled with excellent papers and the attendance was large.

The newly elected officers are: president, Brig. Gen. Robert A. Blood, Surgeon-General of Massachusetts; first vice-president, Medical Director Wise, U. S. Navy; second vice-president, Surgeon-General Walter Wyman, U.S. Marine Hospital Service; treasurer, Lieut. Herbert A. Arnold, Assistant-Surgeon Penn. National Guards; secretary and editor, Major James Evelyn Pilcher, Brigade Surgeon U. S. V., captain, retired, U. S. A.; assistant secretary, Lieut. T. W. Richards, Past Assistant-Surgeon, U.S.N. Major A. H. Briggs, M.D., N.Y.N.G., of Buffalo, is chairman of transportation committee.

The association is in a prosperous condition and has a membership of 700. Representatives of foreign governments attended the meeting, delegates from England, Canada, Mexico, Italy, Austria, France and Japan, being present. The association is about to be incorporated, a bill for that purpose being in the present congress. The next meeting will be held in Boston, in June, 1903.

The American Urological Association held its first annual meeting at Saratoga, June 13 and 14, 1902, under the presidency of Dr. Raymond Guitéras, of New York. The program was an interesting one, beginning with the president's address on Advances in urology. The attendance was large and the meeting was satisfactory in every respect.

---

The Buffalo Academy of Medicine held its annual meeting Tuesday evening, June 17, 1902, at which Dr. Herman E. Hayd was elected president, and Dr. Francis W. McGuire was elected secretary for the current year.

---

The American Academy of Medicine held a large meeting at Saratoga, June 7-9, 1902, at which an interesting program was disposed of. The influence of the academy is increasing year by year. Dr. Charles McIntire, of Easton, was elected president.

---

The Buffalo Academy of Medicine held meetings during the months of May and June as follows:

*Section on Obstetrics and Gynecology.*—Tuesday evening, May 27. Program: Some thoughts connected with breach delivery, P. W. Van Peyma. Complications and degenerations of fibroid tumors, C. C. Frederick. Annual election of officers of this section.

*Annual Meeting.*—Tuesday evening, June 17. Program: Election of officers; reports of officers. An address by the retiring president, Charles G. Stockton. Installation of newly elected officers.

---

The United States Pension Examiners of the State of New York held a meeting at Saratoga Springs, June 9, 1902, at which the following program was observed: (1) Address of welcome, E. Valencourt Deuell, President of Saratoga Board. (2) Defects in reports of examining surgeons, with suggestions for their improvement, J. F. Raub, Medical Referee Pension Bureau, Washington, D. C.; discussion opened by William Warren Potter, Buffalo. (3) The influence and value of organisation, George W. Miles, Oneida; discussion opened by W. L. Ayer, Owego. (4) The advantages of organisation from the point of view of the special examiner, Wheelock Rider, Rochester; discussion opened by John N. Oliver, Brooklyn.

The paper of Dr. Raub attracted great attention, as it dealt with a subject of much importance to the members. It was the sense of the meeting that a National organisation should be perfected and steps were taken to that end. Great credit is due Dr. William A. Howe, of Phelps, for the success of the Saratoga meeting.

